# A Flame-Retardant Phytic-Acid-Based LbL-Coating for Cotton Using Polyvinylamine

**DOI:** 10.3390/polym12051202

**Published:** 2020-05-25

**Authors:** Olga Zilke, Dennis Plohl, Klaus Opwis, Thomas Mayer-Gall, Jochen Stefan Gutmann

**Affiliations:** 1 Deutsches Textilforschungszentrum Nord-West gGmbH, Adlerstrasse 1, D-47798 Krefeld, Germany; dennis.plohl@dtnw.de (D.P.); opwis@dtnw.de (K.O.); mayer-gall@dtnw.de (T.M.-G.); 2 Physical Chemistry & CENIDE, University Duisburg-Essen, Universitätsstrasse 5, D-45117 Essen, Germany

**Keywords:** textiles, cotton, phytic acid, polyvinylamine, flame retardant finishing, layer-by-layer

## Abstract

Phytic acid (PA), as a natural source of phosphorus, was immobilized on cotton (CO) in a layer-by-layer (LbL) approach with polyvinylamine (PVAm) as the oppositely charged electrolyte to create a partly bio-based flame-retardant finish. PVAm was employed as a synthetic nitrogen source with the highest density of amine groups of all polymers. Vertical flame tests revealed a flame-retardant behavior with no afterflame and afterglow time for a coating of 15 bilayers (BL) containing 2% phosphorus and 1.4% nitrogen. The coating achieved a molar P:N ratio of 3:5. Microscale combustion calorimetry (MCC) analyses affirmed the flame test findings by a decrease in peak heat release rate (pkHRR) by more than 60% relative to unfinished CO. Thermogravimetric analyses (TGA) and MCC measurements exhibited a shifted CO peak to lower temperatures indicating proceeding reactions to form an isolating char on the surface. Fourier transform infrared spectroscopy (FTIR) coupled online with a TGA system, allowed the identification of a decreased amount of acrolein, methanol, carbon monoxide and formaldehyde during sample pyrolysis and a higher amount of released water. Thereby the toxicity of released volatiles was reduced. Our results prove that PA enables a different reaction by catalyzing cellulosic dehydration, which results in the formation of a protective char on the surface of the burned fabric.

## 1. Introduction

Textiles are used in a wide variety of applications, including carpets, clothing, upholstery or furnishing fabrics. They are made of synthetic and natural polymers, have a large surface area and an organic origin [1]. Therefore most of them are flammable and thereby potentially hazardous unless suitable protective coatings are applied [2]. In 2017/18 about 25% of household fires were caused by textiles, upholstery and furnishings, while 46% of all fire-related fatalities were associated with these fires [3]. 

In order to avoid ignition and spread of the fire, many textile products are finished with flame retardants (FRs) [1,4,5,6]. Due to their high efficiency, brominated FRs have been used for textile coatings for a long time [1,7]. However, their toxicity and environmental impact together with a potential risk of exposure to humans and the environment, led to legal restriction and prohibition [8,9,10,11]. For that reason, an intense research activity focused on halogen-free alternatives, and various phosphorus- and nitrogen-containing compounds were recently proposed as interesting FRs [12,13,14,15,16,17,18]. Furthermore, the development of bio-based flame-retardant materials made of renewable instead of fossil resources became one of the latest objectives in this field [19]. 

**Figure 1:**
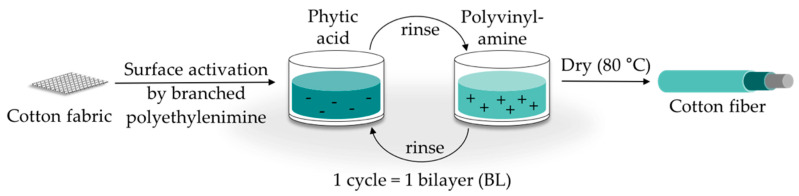
Schematic of LbL assembly using phytic acid and polyvinylamine. The cycles are repeated until the desired number of bilayers is obtained.

Phytic acid (PA) is a low molecular weight antinutrient [20] and has a strong chelating ability to form cation-phytate complexes [21]. In plant seeds and grains, phytate is present as the main phosphorus storage form [22]. O’Dell et al. [23] found the major proportion of all analyzed elements in the outer layers of wheat and rice kernels whereas phytates were mainly found in the germ. Since these parts are removed by milling during cereal grain processing [23], phytate can be produced from these food industry waste products [24]. Several studies have indicated that PA can reduce textile flammability [25,26,27,28]. This is due to the ability of acidic phosphorus-based FRs to enhance the cellulosic carbonization by dehydration and to decrease the release of flammable gases [29,30,31]. In combination with nitrogen-compounds, phosphorus-based FRs can improve their efficiency by a P-N-synergism on cellulose [32,33,34]. Initial findings, summarized by Horrocks [35], suggested that nitrogen forms polymeric compounds containing P-N bonds by nucleophilic attack on the phosphate group. In the study of Cheng et al. [25] PA was used for the synthesis of a phosphorus-based FR which was covalently linked to wool. They investigated a self-extinguishing effect in the vertical flame test after 20 washing cycles. As an alternative to covalent linkage, layer-by-layer (LbL) assembly is another strategy of textile surface modification by PA due to its highly charged phosphate groups [36,37]. LbL assembly involves alternate deposition of positively and negatively charged electrolytes to build multilayered films on the substrate (Figure 1) [38,39,40]. Laufer et al. [26] immobilized PA and chitosan as an environmental-friendly flame-retardant finishing for cotton (CO) by LbL technique. The composition of the coating was influenced by varying the pH of the deposition solutions. Fabrics coated with 30 bilayers (BL) exhibited a self-extinguishing effect in a vertical flame test. 

For LbL coatings water can be used as solvent, no additional crosslinking agent is required and the deposition solution can be recycled [41]. These characteristics make the process sustainable. 

Given this background, our work focuses on the immobilization of phosphorus-containing PA on CO using an LbL approach with polyvinylamine (PVAm) to create a partially bio-based flame-retardant finish. Since phosphorus- and nitrogen-containing FRs often act as flame-retardant synergists, a high nitrogen content potentially leads to a favorable flame-retardant performance. PVAm contains the highest amount of primary amine groups of all polymers with a nitrogen content of 32.5%, whereas chitosan as a biopolymer alternative has 8.7% nitrogen content. Hence, the commercially available PVAm acts as a cationic polyelectrolyte and synthetic nitrogen-rich source in our coating. 

Stable ion pairs were generated by LbL assembly and varying number of BL. Flame-retardant properties were investigated according to ISO 15025:2016. In addition, the thermal stability was examined by means of thermogravimetric analysis (TGA) and microscale combustion calorimetry (MCC). The surface topography was studied by use of scanning electron microscopy (SEM). Fourier transform infrared spectroscopy (FTIR) was employed for chemical analysis of fabrics before and after flame test. TGA coupled with a FTIR spectrometer was used for gas phase investigations.

## 2. Materials and Methods 

### 2.1. Materials

Woven CO fabric (plain weave, 170 g/m^2^, white) was purchased from wfk Testgewebe GmbH, Brüggen, Germany. PA (50% in water) was obtained from TCI Deutschland GmbH, Eschborn, Germany. Technical PVAm, sourced from Lupamin^®^ 9095 (L9095, >90% degree of hydrolysis, solid content 20–22%), and branched polyethylenimine (BPEI), sourced from Lupasol^®^ WF (99% BPEI), were purchased from BASF. 

Nitric acid (69%, supra-quality ROTIPURAN^®^, Carl Roth GmbH + Co. KG, Karlsruhe, Germany) was used for microwave-assisted digestion of textile samples for phosphorus determination by inductively coupled plasma optical emission spectroscopy (ICP-OES).

For the potentiometric determination of Kjeldahl nitrogen H_2_SO_4_ (ROTIPURAN^®^ 98%), NaOH (50%, extra pure) and HCl (0.05 mol/L, volumetric solution) were purchased from Carl Roth GmbH+ Co. KG, Karlsruhe, Germany, H_3_BO_3_ (≥99.5%, Ph.Eur., USP, BP) from Bernd Kraft GmbH, Duisburg, Germany and Kjeltabs Auto, used as a digestion catalyst, from Thompson and Capper Ltd, Runcorn, UK. 

### 2.2. Instrumentation

ICP-OES spectra were recorded using a Varian (Agilent) 720-OES (Varian Inc, Darmstadt, Germany). Samples for ICP-OES measurements were digested using a Mars Xpress instrument (CEM GmbH, Kamp-Lintfort, Germany). For Kjeldahl nitrogen determination the digestion unit TURBOTHERM^®^ and the steam distillation system VAPODEST^®^ were obtained from C. Gerhardt GmbH & Co. KG, Königswinter Germany and the automatic titration system from Deutsche Metrohm GmbH & Co. KG, Filderstadt, Germany. TGA analyses were performed by means of Discovery TGA 55 (TA Instruments, Hüllhorst, Germany) and coupled with a FTIR spectrometer (IRPrestige-21 Shimadzu Deutschland GmbH, Duisburg, Germany). The samples were dried for 48 h at 60 °C prior to analysis. Measurements were conducted under nitrogen and air (90 mL/min) in a platinum crucible with samples weighing (10 ± 0.1) mg. The system was first allowed to equilibrate at 40 °C and isothermally held at this temperature for 5 min. Samples were then heated from 40 °C at a rate of 20 K/min to 800 °C where it was held for 10 min before cooling down. Measurements were performed using a FAA Micro Calorimeter (Fire Testing Technology, East Grinstead, UK) following ASTM D 7309 Method A. The samples were pyrolyzed under N_2_ with a heating rate of 1 K/s to 750 °C and then combusted at 900 °C in a mixture of O_2_/N_2_ (20:80). The recorded data were analyzed using Microcal Origin 2018b Professional software. SEM (S-3400N II, Hitachi High-Technologies Europe, operating at an accelerating voltage of 10 kV) and attenuated total reflection (ATR)-FTIR (Specac Ltd, Orpington, UK) equipped with a diamond crystal, with a resolution of 4 cm^−1^, were used for morphological and chemical characterization of fabrics before and after flame tests. Flame tests were conducted according to ISO 15025:2016, Method A and B. Passing requirements were assessed following ISO 11611:2015. 

### 2.3. LbL Assembly

All reagents were used as received. Demineralized water (DW, 1.8 MΩ, pH ~ 6.8) was used to prepare deposition solutions. A 5% PA solution (pH ~ 0.7) and 5% L9095 solution (pH ~ 8.7) were used for the coating of the textiles. The deposition procedure is shown in Figure 1. First, the textile surface was activated using a 1% BPEI solution (pH ~ 11.7), according to Laufer et al. [26]. Then the substrates were alternately dipped into the PA and L9095 solution. The first immersion steps lasted for 5 min each, subsequent steps 1 min. Each immersion step was followed by rinsing with DW (2 min) to remove unbonded residues. Subsequently, textiles were wrung out. After the desired number of BL was obtained, the samples were dried at 80 °C. 

### 2.4. Characterization of Immobilized Compounds

The phosphorus content of finished textiles was quantitatively determined using ICP-OES of 200 mg samples digested with 8 mL HNO_3_ in a microwave. The samples were then diluted with ultrapure water to a volume of 25 mL, filtered and analyzed. 

The mass add-on A (%) was calculated using

(1) $$A=\frac{m_{1}-m_{0}}{m_{0}}\times 100\%$$

where m_0_ is the mass before treatment (g) and m_1_ is the mass after treatment (g). 

Nitrogen content was determined by potentiometric Kjeldahl titration. For that reason, the samples (200 mg) were digested in 20 mL sulfuric acid. Then, during the steam distillation NaOH was added to the digested sample. The formed ammonia was distilled off and transferred into the receiver vessel with aqueous H_3_BO_3_ solution as absorbing solution. The captured ammonium ions were determined by potentiometric titration with HCl.

## 3. Results and Discussion

### 3.1. Coating Properties

Many phosphorus- and nitrogen-containing FRs in cellulose are able to promote char formation [42]. That was revealed by detailed studies of formed char, which showed that most phosphorus-containing FRs were not volatilized, but remained in the char instead [43]. An increase in the flame-retardant content, enhances the char formation and thus the flame-retardant characteristics of the coating. However, the effectiveness of phosphorus compounds differs from each other, depending on their chemical structure. Hendrix and coworkers [14] suggested that a phosphorus content of at least 1.5% (*w*/*w*) is required for the finishing to be efficient. Later studies by Tesoro [44] found a minimum of 2–4% (*w*/*w*) phosphorus content, whereas some nitrogen compounds were able to decrease the minimum phosphorus amount required. For that reason, nitrogen-rich polymers such as PVAm can be used to achieve better flame-retardant properties. The synergism of phosphorus- and nitrogen-containing FRs in cellulose can be explained by an enhancement of phosphorylation of cellulose and an increased charring and gas phase mode of action [35,45]. PA has a phosphorus content of about 28% (*w*/*w*), therefore add-ons of 7%–14% (*w*/*w*) are needed to achieve phosphorus levels of 2%–4% (*w*/*w*). 

Results of determined phosphorus and nitrogen mass fractions x, the molar P:N ratio as well as the mass add-on are summarized in Table 1. As expected, add-on increases linearly with increasing number of BL. After deposition of 15 BL the phosphorus content was up to 2% and the nitrogen content 1.4%. Considering the synergistic effect of phosphorus and nitrogen in flame-retardant finishings and a required phosphorus content of at least 1.5%, samples with 10 and 15 BL are expected to have good flame-retardant properties. Furthermore, several studies, reviewed by Heywood et al. [46], have shown that the P:N molar ratio influences the flame-retardant performance of the coating. Our samples coated with 15 BL had a P:N molar ratio of ~3:5. 

### 3.2. Thermal Stability

TGA and MCC were used for the evaluation of the thermal stability and the combustion behavior of coated CO fabrics. Nitrogen atmosphere in TGA was employed to assess the non-oxidative thermal decomposition behavior of the condensed phase and air was used for oxidation studies. For gas phase analyses, TGA was coupled online with a FTIR spectrometer. Results of TGA-FTIR measurements in nitrogen are shown in Figure 7 and results obtained in air are available in Supplementary Materials. The materials’ combustibility was analyzed by MCC measurements, where pyrolysis products are mixed with access of oxygen/nitrogen under constant thermal conditions to determine the heat release rate (HRR) based on the oxygen consumption. Despite of differences in the heating rates used in MCC (1 K/s) and TGA (0.3 K/s) measurements under nitrogen, the decomposition of our uncoated and coated CO fabrics started at the same temperature, the number of decomposition steps as well as the decomposition peak temperature and the remaining char were similar.

Cellulosic pyrolysis proceeds in two competing reactions. In the first reaction, a char is formed after dehydration [47]. In the second, volatile flammable products are released after depolymerization to levoglucosan [47]. To promote the formation of a protective char, FRs need to catalyze cellulosic dehydration at temperatures lower than 300 °C [48]; P-containing FRs are able to induce these reactions [41,49]. In air, the maximum rate of weight loss emerges earlier than in nitrogen [50] and thermal degradation occurs mainly by oxidation reactions leading to the evolution of water, carbon monoxide and carbon dioxide [47]. 

**Figure 2:**
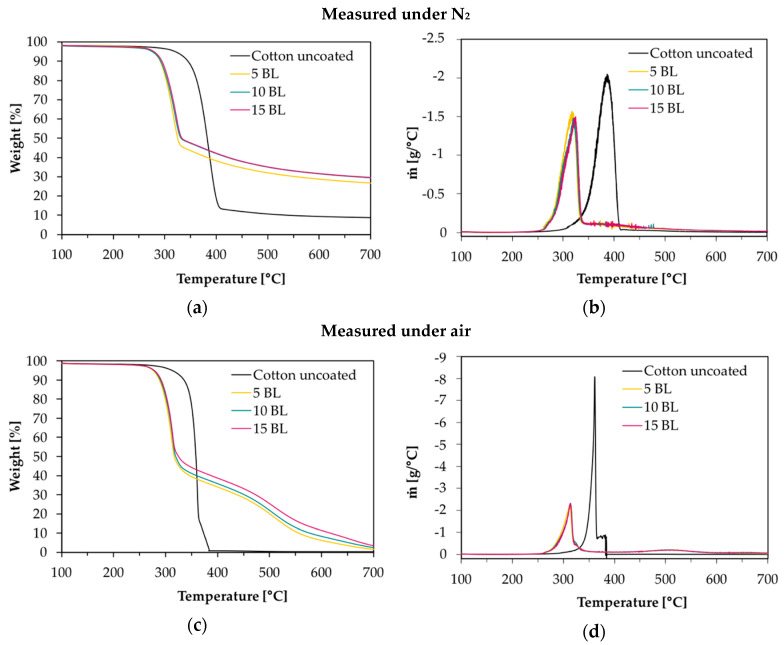
Thermogravimetric analysis (TGA) curves of coated and uncoated cotton fabrics, measured under nitrogen and air with 20 K/min heating rate. (**a**) TGA curves in nitrogen; (**b**) differential TG (DTG) curves in nitrogen; (**c**) TGA curves in air; (**d**) overlapping DTG curves in air.

The TGA and corresponding derivative weight loss curves of the coated samples are shown in Figure 2. Table 2 presents the related data. All fabrics show a similar degradation behavior with one decomposition step in nitrogen and two decomposition steps in air. Compared to uncoated CO, however, coated fabrics exhibit a significantly decreased decomposition temperature, measured at 5% weight loss (T_∆5%_), while the corresponding residues (Res) of all finished fabrics increased (Figure 2a,c). This implies that a char, which was thermally stable up to 700 °C, was formed. The maximum in the differential TG curve (T_max_) was shifted from 388 to 324 °C in nitrogen and from 362 to 314 °C in air (Figure 2b,d). Comparing measurements in nitrogen and air, the shift of T_max_ of uncoated CO and the increased mass loss rate ṁ at T_max_(CO) obtained in air, proves that oxygen plays a major role in accelerating cellulosic decomposition reactions (Figure 2b,d) [50,51]. Contrary to the strong rise of ṁ for CO in air, ṁ of coated fabrics was slightly increased from −1.4 to −2.2 g/°C (Figure 2b,d). 

Under nitrogen the remaining residue was increased by a factor of about 3 compared to uncoated CO. Since char formation in flaming combustion proceeds under anaerobic conditions, our results obtained in nitrogen prove that coated fabrics have good char formation properties with high char yields. In air, the char residue was enhanced by a factor of ~10. Hence, in both nitrogen and air, coated fabrics degrade slower with a lower mass loss rate leaving a higher solid residue behind. 

**Figure 3:**
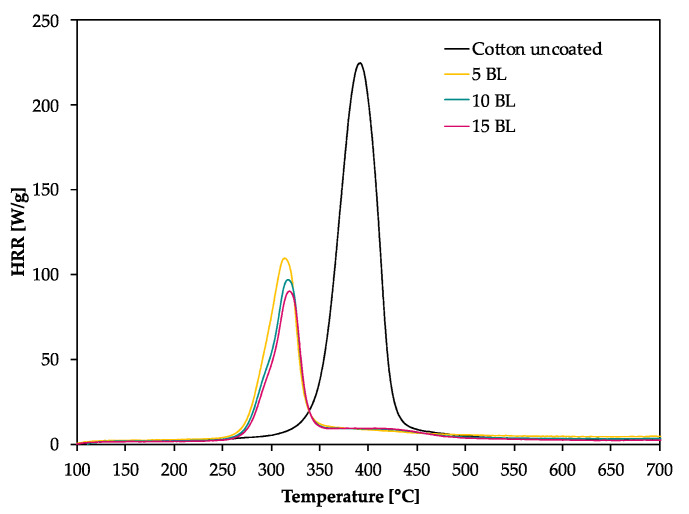
Microscale combustion calorimetry (MCC) measurements of coated and uncoated cotton fabrics, measured under nitrogen with 1 K/s heating rate.

Figure 3 depicts the effect of the coating on the HRR measured in the MCC. Similar to TGA results (obtained under nitrogen), all fabrics decompose in one step. With the increasing number of BL, the peak of HRR (pkHRR) decreases by more than 60%. In addition, the shift of the temperature of pkHRR (T_pkHRR_) indicates that the flame-retardant coating makes an impact on the decomposition of CO. This may be explained by the presence of PA as a dehydrating agent. Results of MCC measurements are given in Table 3 and the complete data set is shown in Appendix A Table A1. Three curves were fitted for 15 BL due to the curve fitting procedure. For a good fitting, two peaks are needed to fit the decomposition signal at around 300 °C. A second broad decomposition signal, also present in differential TG (DTG) curves (Figure 2b), is seen at around 400 °C. That signal could be explained by a slow release of decomposition products due to the formed isolating char. To determine the heat release capacity (HRC), the peak maxima around 300 °C and 400 °C were summed up.

All finished textiles exhibit a reduced HRC and total heat release (THR). The amount of char production was increased from 8.9% for uncoated CO to 32.3% for finished CO (15 BL). The decreasing THR is explained by the enhanced char formation which results in a lower amount of released combustible gases. As a consequence, less oxygen is consumed in the combustion chamber of the MCC and thereby less energy can be released. TGA results confirm these findings by a reduction in ṁ in the DTG curve by about 41% (Figure 2b). 

Coated fabrics exhibit a broad signal with a temperature of pkHRR (T_pk1HRR_) at around 400 °C. This temperature corresponds to the T_pk1HRR_ of uncoated CO, however, with a much lower pkHRR. Such behavior is indicative of a protective barrier formed in the first reactions via P–N bonds and phosphorylation of cellulose [35]. This barrier acts in the second decomposition signal, so that fewer flammable cellulosic degradation products, thus less energy, is released. Furthermore, the barrier strongly influences the char residue. Since the samples are pyrolyzed in a local atmosphere with high nitrogen content, sample oxidation does not occur. Hence, the residue of uncoated CO is higher than in air [50]. Yeh and Barker [43] reported that most phosphorus of flame-retardant cellulose remains in the char. They also found that an increase in phosphorus is related to an enhanced char formation. The fact that the char residue of coated fabrics is higher than the mass add-on of the coating, proves that such an insulating layer was formed (Table 4).

As mentioned above, cellulose thermally degrades in two competing reactions. In one reaction proceeding at lower temperatures (200 to 280 °C), a char is formed after dehydration, depolymerization, hydrolysis, oxidation and decarboxylation [35,52]. In the other reaction at higher temperatures (280 to 340 °C), levoglucosan is formed [35,50]. Further pyrolysis of levoglucosan results in the release of highly flammable, mostly low molecular weight products [35,47]. The formation of levoglucosan can be reduced in the presence of acidic compounds, like PA. In general, by adding phosphorus compounds, phosphoric acid is formed on heating and thereby cellulosic dehydration is catalyzed at lower temperatures [53]. In combination with nitrogen compounds, more complex reactions are possible. Polymeric P-N bonds are formed, which increase the ability to phosphorylate cellulosic hydroxyl groups [35]. In this way, the formation of levoglucosan is inhibited by the P/N-containing FR, fewer flammable volatiles are released, and the protective char prevents the material from further degradation. We presume that an increase in the mass add-on would lead to a stronger decreased pkHRR. The contribution of PA or PVAm individually and combined as synergists to an additional flame-retardant effect would therefore be interesting.

### 3.3. Flame Retardant Properties

Flame tests provide information about the flammability of the coated material. Depending on the textile application area, different standards for the textile flammability assessment can be used. ISO 15025:2016, which is specified for protective clothing, was chosen as a suitable test. Sample sizes were (200 × 160) mm for surface and (200 × 80) mm for bottom edge ignition, according to the standard. The samples were not conditioned prior to testing and the test results were characterized according to the pass or no-pass criteria specified in ISO 11611:2015 (protective clothing for use in welding and allied processes). 

**Figure 4:**
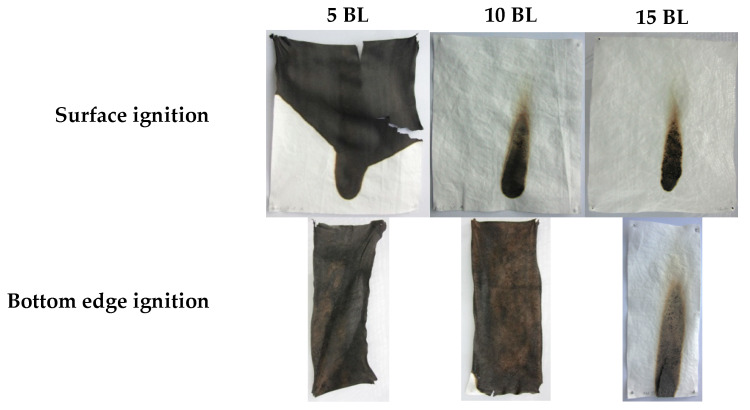
Coated fabrics (5, 10, 15 BL) after the flame tests, according to ISO 15025:2016 Method A and B, of surface and bottom edge ignition. Uncoated cotton fabrics burned completely down.

Figure 4 shows differently coated fabrics after the flame test with surface and bottom edge ignition, respectively. Untreated CO fabrics burned completely down. In all cases a protective layer was formed while the fabric structure remained unchanged. 

Table 5 lists the evaluation of the flame test results. No after flame time was observed from 10 BL (surface ignition). For bottom edge ignited fabrics, 15 BL were needed to reduce the afterflame time to zero. All treated fabrics exhibited a much lower burning and afterglow time was reduced to zero for all samples. Regarding the phosphorus content (Table 1), a minimum of 1.4% (surface ignition) and 2.0% (bottom edge ignition) was necessary to pass the requirements of ISO 11611:2015.

**Figure 5:**
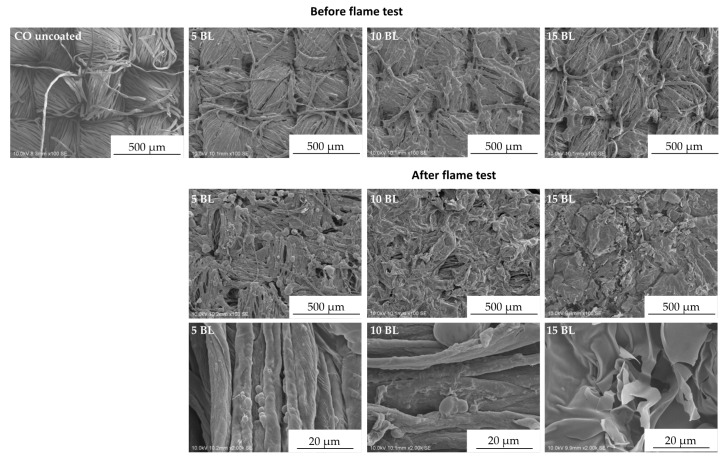
Scanning electron microscopy top view images of untreated, treated and burned cotton fabrics. Top row: fabrics before flame test; middle and bottom row: charred samples after flame test in different magnifications.

The burning behavior of coated fabrics can be described referring to MCC and TGA results. These results revealed a lower ṁ (TGA) and THR (MCC). Furthermore, the T_pk1HRR_ measured in the MCC and the residue at the peak temperature T_max_ (TGA), shown in Table 2, have clearly changed. The shifted T_pk1HRR_ and the decreased HRC have a positive effect on the flame-retardant properties of the coated fabrics. A decreased ṁ (TGA) proves that fewer combustible gases are released into the gas phase. Due to a decreased release of fuel gases heat production was reduced resulting in lower THR, T_pk1HRR_ and pkHRR values. A shifted T_pk1HRR_ indicates that the dehydration reaction of cellulose, caused by phosphorus catalysis, occurs at lower temperatures. The carbonized layer is formed and thus shields the combustible material below. In this way the fire spread can be inhibited or delayed. These changes were confirmed in the flame tests (Figure 4) and the SEM study (Figure 5), as in all cases the textile structure remained unchanged. HRC values can also be correlated to a UL 94 flame test, as suggested by Lyon et al. [54]. With a HRC lower than 100 J/(g K), our samples coated with 10 and 15 BL could achieve a UL 94 V0 rating or better (no ignition, no afterflame).

The surface morphologies of untreated, coated and burned fabrics were investigated by SEM (Figure 5). Compared to uncoated CO, the surface of coated fabrics was changed by dispersion of the flame-retardant coating on and around the fiber, without affecting the textile structure.

Contrary to 10 and 15 BL, samples finished with 5 BL formed blisters on the surface after burning. For 10 BL blisters were found on fibers exposed by gaps in the char layer (see high magnification images in Figure 5, 10 BL). Phosphorus and nitrogen compounds are able to catalyze the dehydration of CO [41,49] and can act in both, gas and condensed phase [55,56]. It may be assumed that the blisters form as a result of gases formed during the thermal decomposition of PVAm and PA on CO. 

The high-magnification SEM images demonstrate that all burned fabrics exhibit a protective char layer that shields the material from heat and oxygen and prevents it from further degradation. The char formation increases with the increasing number of BL. However, in comparison to 15 BL, the surface of fabrics coated with 5 and 10 BL are not homogeneously covered with the char. The according “gaps” in the char layer still expose the fabric structure, which may allow heat, mass and oxygen transfer and thereby decrease the flame-retardant properties. This is consistent with the flame test results shown in Figure 4, where only 15 BL passed the flame test requirements of ISO 11611:2015 in both surface and bottom edge ignition tests. 

### 3.4. Char and Gas Phase Analysis

**Figure 6:**
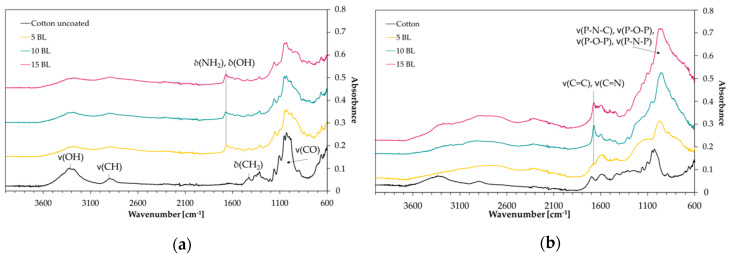
ATR-FTIR absorbance spectra. (**a**) Uncoated and coated cotton samples; (**b**) charred samples after flame test.

ATR-FTIR-spectra of coated and charred samples after the flame test are shown in Figure 6. Depending on the polymorphic form of cellulose, intensity and positions of O-H stretching vibration bands can vary. We found one strong band at 3319 cm^−1^ (Figure 6a). CH stretching appears around 2885 cm^−1^. Deformation vibrations of CH_2_ occurred near 1421 cm^−1^ and several absorption bands in the fingerprint area around 1028–1323 cm^−1^ can be assigned to characteristic CO peaks. Band intensities of coated fabrics decrease due to new functional groups on the surface. A significant broad band near 1672 cm^−1^ appears due to the –NH_2_ and the OH (in P=O(OH)) deformation vibrations. The IR-spectra proved that the coating changed the fabrics surface. On charred samples (Figure 6b) the intensity of the band at 1672 cm^−1^ was increased, which is assigned to C=C (in P-C=C or alkenes) double bond or C=N stretching vibrations. These are formed during solid phase reactions at high temperatures, resulting in carbonaceous surface structures by cyclization and crosslinking of double bonds. The intensity of the broad band around 940 cm^−1^ increases with the number of BL. P–O–P, P–N–C, P–N–P and P–O–C stretching can be assigned to this signal [57,58,59]. These findings prove that a protective char layer was formed during the flame test. In combination with the results of MCC and TGA measurements, where high char yields were obtained, we conclude that a high phosphorus content remained in the char. 

**Figure 7:**
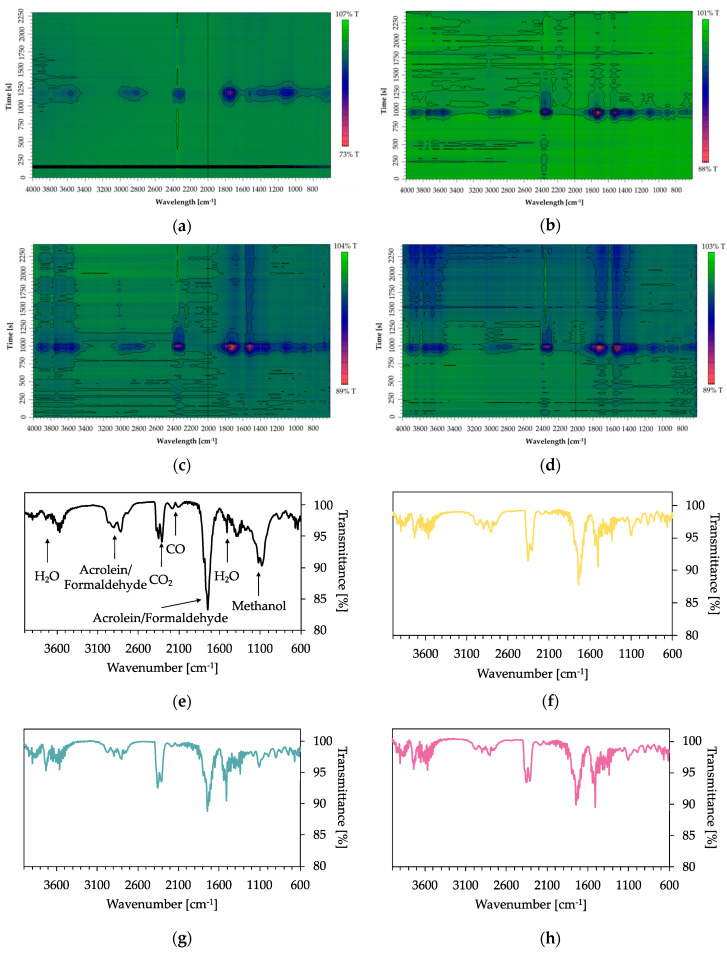
2D plots and FTIR-spectra of the corresponding peak maxima of uncoated and coated cotton fabrics, obtained by TGA-FTIR investigations in nitrogen. (**a**) 2D plot of uncoated cotton; (**b**) 2D plot of 5 BL; (**c**) 2D plot of 10 BL; (**d**) 2D plot of 15 BL; (**e**) spectrum at 1180 s (393 °C in TGA) of uncoated cotton; (**f**) 5 BL spectrum at 970 s (323 °C in TGA); (**g**) 10 BL spectrum at 1000 s (333 °C in TGA); (**h**) 15 BL spectrum at 980 s (327 °C).

TGA-FTIR investigations provide information about the identity of released gaseous compounds and the time dependence of gas evolution. Spectra of absorbance maxima obtained from Gram–Schmidt profiles are presented in Figure 7e–h. Associated TGA measurements in nitrogen are shown in Figure 2a,b. The peak shift of T_max_ (TGA) corresponds to the time peak shift in the 2D plots of coated fabrics (Figure 7a–d). In Figure 7a, the first peak maximum (125 s) of uncoated CO is an artefact. The second peak maximum, at 1180 s, has an absorbance of 7.6. Since fewer volatile compounds can be released by the coated pyrolyzing samples, absorbance successively decreases from 4.8 for 5 BL (b), 3.6 for 10 BL (c) to 3.2 for 15 BL (d). 

In the spectra shown in Figure 7e–h, the strongest signals were found at 1735 cm^−1^ and 1710 cm^−1^, which are assigned to cis and trans C=O stretching vibrations of acrolein. Acrolein is a flammable and toxic compound by all exposure routes. Furthermore, the band at 1735 cm^−1^ appears due to the C=O stretching of formaldehyde. CH_2_ stretching vibrations of formaldehyde at 2800 cm^−1^ were found, its intensity decreased with the flame-retardant coating. The release of CO_2_ (2349 cm^−1^) and water (~1500 cm^−1^) of the coated fabrics increase in comparison with uncoated fabrics, while the evolution of carbon monoxide (stretching vibrations at 2171 cm^−1^) decreases. Transmittance of C-O stretching vibrations around 1070 cm^−1^ was enhanced with growing number of BL. This band reflects methanol evolution. 

On basis of FTIR char analyses and TGA-FTIR results we conclude that organic components were bound in the char as a result of a PA-induced dehydration reaction leading to a reduced release of fuel gases (volatile compounds). The reduction in the released flammable and toxic pyrolysis products proves the effectiveness of the flame-retardant coating. 

## 4. Summary

Phytic acid and polyvinylamine bilayers were deposited on cotton fabrics using layer-by-layer assembly. Increasing the number of bilayers resulted in an increasing phosphorus and nitrogen content. The finishing led to a flame-retardant behavior (no afterflame and afterglow time, no ignition). Cotton finished with 15 bilayers—with a mass add-on of around 19%, a phosphorus content of 2% and a nitrogen content of 1.4%—passed the bottom edge ignition flame test according to ISO 15025:2016. Microscale combustion calorimetry and thermogravimetric analysis (TGA) measurements showed a shift of the decomposition temperatures in combination with high char yields. From Fourier transform infrared spectroscopy (FTIR) and scanning electron microscopy analyses, cellulosic dehydration by phytic acid as an acid catalyst and the formation of a protective char layer, as a physical barrier on the surface of the fabric, were proven. More water was released during pyrolysis in the TGA-FTIR investigations confirming the assumption of acid catalyzed dehydration. The lower carbon monoxide, formaldehyde, methanol and acrolein release, shown by TGA-FTIR, proves that the flame-retardant coatings also reduce the toxicity of the pyrolysis gases.

In conclusion, we achieved excellent flame-retardant properties of cotton fabrics with a coating of 15 bilayers containing polyvinylamine, which is produced on industrial scale, and phytic acid as a grain processing by-product. With our partially bio-based coating fewer procedure steps are needed to accomplish the same effect as a chitosan/phytic acid coating, requiring 30 bilayers [26]. However, flame-retardant textile finishes usually have to fulfill challenging parameters, e.g., durability and applicability [60]. These requirements can limit the industrial usage as layer-by-layer coatings are mostly non-durable and time- and labor-intensive [60,61]. Hence, Chang et al. [61] developed a continuous layer-by-layer deposition process proving the layer-by-layer scale-up viability. For industrial and technical applications future studies will be necessary to investigate textile properties such as the washing stability of the coating, the tensile strength of the fabric and the scale-up applicability. 

## Figures and Tables

**Table 1:** Summary of determined mass add-on, phosphorus- and nitrogen-mass fractions *x* and molar P/N ratio of coated cotton fabrics.

Sample	Mass Add-On %	*x*_P_ Measured ^1^ %	*x*_N_ Measured ^2^ %	P/N Molar Ratio mol/mol
5 BL	5.8 ± 0.2	0.8 ± 0.04	0.7 ± 0.01	1:2
10 BL	11.6 ± 0.9	1.4 ± 0.08	1.0 ± 0.03	3:5
15 BL	18.8 ± 2.1	2.0 ± 0.08	1.4 ± 0.13	3:5

^1^ Measured quantitatively by ICP-OES. ^2^ Measured quantitatively by Kjeldahl nitrogen determination.

**Table 2:** Thermogravimetric analysis data, measured under nitrogen and air.

Sample	T_∆5%_ °C	T_max_ (Res) °C (%)	Res (700 °C) %
		**Measured under N_2_**	
CO uncoated	322 ± 2	388 ± 1 (41.0 ± 2.0)	8.7 ± 0.2
5 BL	280 ± 1	321 ± 1 (58.2 ± 1.3)	24.1 ± 1.0
10 BL	278 ± 1	323 ± 1 (58.4 ± 1.2)	28.2 ± 1.1
15 BL	279 ± 1	324 ± 1 (59.8 ± 0.9)	29.1 ± 0.9
		**Measured under air**	
CO uncoated	311 ± 1	362 ± 1 (31.1 ± 0.2)	0.3 ± 0.1
5 BL	277 ± 1	313 ± 1 (57.7 ± 0.8)	1.6 ± 0.2
10 BL	277 ± 1	313 ± 1 (59.6 ± 1.3)	2.5 ± 0.1
15 BL	278 ± 1	314 ± 1 (60.5 ± 1.0)	3.1 ± 0.3

**Table 3:** Microscale combustion calorimetry measurement data.

Sample	Char %	HRC J/(g K)	THR kJ/g	pk1HRR W/g	pk1HR kJ/g	T_pk1HRR_ °C
CO uncoated	8.9 ± 0.3	216.6 ± 5.7	10.7 ± 0.1	216 ± 5.7	10.7 ± 0.1	387 ± 1
5 BL	28.9 ± 0.7	103.1 ± 4.0	4.9 ± 0.1	94.0 ± 5.5	3.5 ± 0.2	311 ± 2
10 BL	31.3 ± 1.1	90.6 ± 5.8	3.0 ± 0.3	81.5 ± 5.9	3.0 ± 0.3	314 ± 1
15 BL	32.3 ± 1.0	99.2 ± 11.6	4.2 ± 0.2	42.8 ± 5.1	1.8 ± 1.1	297 ± 2

**Table 4:** Comparison between mass add-on and obtained char after TGA and MCC analyses.

Sample	Mass Add-On %	Res (700 °C) (TGA, N_2_) %	Res (700 °C) (TGA, Air) %	Char (MCC) %
CO uncoated	0	8.7 ± 0.2	0.3 ± 0.1	8.9 ± 0.3
5 BL	5.8 ± 0.2	24.1 ± 1.0	1.6 ± 0.2	28.9 ± 0.7
10 BL	11.6 ± 0.9	28.2 ± 1.1	2.5 ± 0.1	31.3 ± 1.1
15 BL	18.8 ± 2.1	29.1 ± 0.9	3.1 ± 0.3	32.3 ± 1.0

**Table 5:** Flame test results of coated cotton samples according to ISO 15025:2016. The stated rating—pass, no pass—is based on two criteria specified in ISO 11611:2015.

Sample	Surface Ignition			Bottom Edge Ignition		
	Afterflame Time	Flame Reaches Upper or Lateral Edge	Test Passed	Afterflame Time	Flame Reaches Upper or Lateral Edge	Test Passed
CO uncoated	Burns completely down within 64 s	yes	no	Burns completely down within 17 s	yes	no
5 BL	57 s	yes	no	18 s	yes	no
10 BL	0 s	no	yes	14 s	yes	no
15 BL	0 s	no	yes	0 s	no	yes

## Supplementary Materials

The following are available online at https://www.mdpi.com/2073-4360/12/5/1202/s1, Figure S1: 2D plots and FTIR-spectra of the corresponding first peak maxima of uncoated and coated cotton fabrics, obtained by TGA-FTIR investigations in air. (a) 2D plot of uncoated cotton; (b) 2D plot of 5 BL; (c) 2D plot of 10 BL; (d) 2D plot of 15 BL; (e) spectrum at 1070 s (357 °C in TGA) of uncoated cotton; (f) 5 BL spectrum at 950 s (317 °C in TGA); (g) 10 BL spectrum at 970 s (323 °C in TGA); (h) 15 BL spectrum at 950 s (317 °C).

## Appendix A

**Table A1:** Complementary microscale combustion calorimetry data set.

Sample	HRC J/(g K)	pk2HRR W/g	pk2HR kJ/g	T_pk2HRR_ °C	pk3HRR W/g	pk3HR kJ/g	T_pk1HRR_ °C
CO uncoated	215.7 ± 5.7						
5 BL	103.1 ± 4.0	9.1 ± 1.9	1.3 ± 0.2	364 ± 5			
10 BL	90.6 ± 5.8	9.1 ± 1.6	1.4 ± 0.1	370 ± 6			
15 BL	99.2 ± 12.0	42.8 ± 5.1	1.5 ± 0.6	324 ± 2	9.0 ± 0.5	1.2 ± 0.1	416 ± 16
