# Supplementary material for: A Flame-Retardant Phytic-Acid-Based LbL-Coating for Cotton Using Polyvinylamine
Source: Polymers (Basel). 2020 May 25;12(5):1202. doi: 10.3390/polym12051202 (PMC7284457; doi:10.3390/polym12051202)
Supplement: Supplementary file 1 [file polymers-12-01202-s001.pdf]

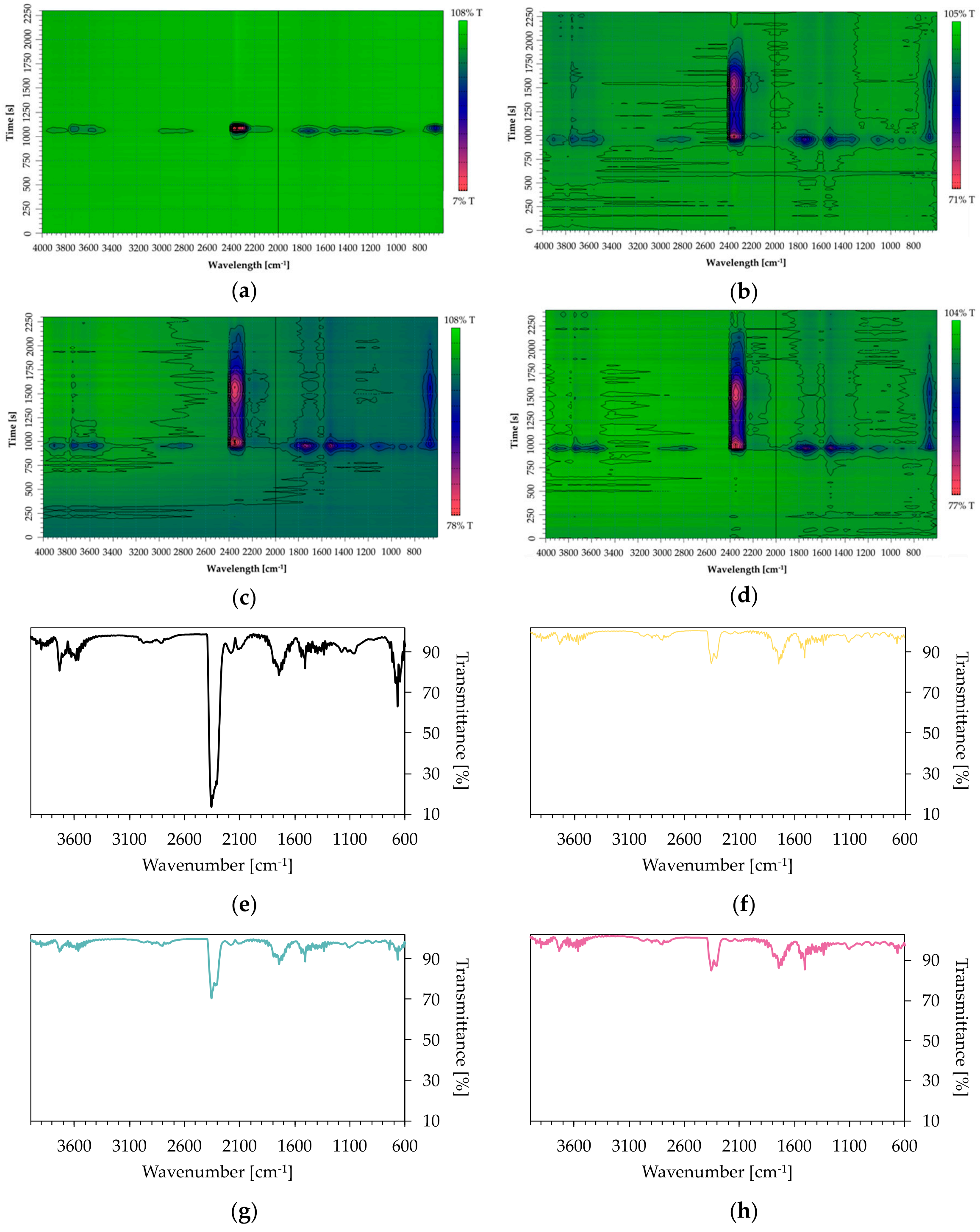

Figure S1. 2D plots and FTIR-spectra of the corresponding first peak maxima of uncoated and coated cotton fabrics, obtained by TGA-FTIR investigations in air. (**a**) 2D plot of uncoated cotton; (**b**) 2D plot of 5 BL; (**c**) 2D plot of 10 BL; (**d**) 2D plot of 15 BL; (**e**) spectrum at 1070 s (357 °C in TGA) of uncoated cotton; (**f**) 5 BL spectrum at 950 s (317 °C in TGA); (**g**) 10 BL spectrum at 970 s (323 °C in TGA); (**h**) 15 BL spectrum at 950 s (317 °C).
